# Sleeping problems in Chinese illicit drug dependent subjects

**DOI:** 10.1186/s12888-015-0409-x

**Published:** 2015-02-19

**Authors:** Jinsong Tang, Yanhui Liao, Haoyu He, Qijian Deng, Guanbai Zhang, Chang Qi, Hangtao Cui, Bin Jiao, Mei Yang, Zhijuan Feng, Xiaogang Chen, Wei Hao, Tieqiao Liu

**Affiliations:** 1 Department of Psychiatry, Institute of Mental Health, the Second Xiangya Hospital of Central South University, 139 Renmin (M) Rd, Changsha, Hunan 410011 P. R. China; 2 Yunnan Institute for Drug Abuse, Kunming, Yunnan China; 3 Department of Psychiatry, Hunan Brain Hospital, Changsha, Hunan China; 4 Department of Neurology, Xiangya Hospital of Central South University, Changsha, Hunan China; 5 School of Public Health, Central South University, Changsha, Hunan China; 6 National Technology of Institute of Psychiatry, Central South University, Changsha, Hunan China; 7 The State Key Laboratory of Medical Genetics, Central South University, Changsha, Hunan China

**Keywords:** Sleep problems, Sleep quality, Illicit drug dependent subjects, Self-reported survey

## Abstract

**Background:**

Illicit drug use/dependence has been recognized as a major problem. Clinical studies demonstrate that poor sleep quality is associated with increased frequency of drug use and relapse. However, few studies have addressed the issue of sleep quality among illicit drug dependent subjects.

**Methods:**

This cross-sectional study explored sleep quality in drug dependent subjects in China. We studied 2178 illicit drug dependent subjects from drug rehabilitation centres in Changsha and 2236 non-drug-using subjects, all of whom completed the self-report Pittsburgh Sleep Quality Index (PSQI).

**Results:**

We found that the prevalence of sleep disturbance was much higher in drug users (68.5%, PSQI >5; specifically, 80.24% in heroin users, 54.16% in methamphetamine users and 81.98% in ketamine users with PSQI >5) than non-users (26.4%, PSQI >5). Drug users had approximately twice the sleep latency than nondrug users (37.7 minutes V.S 18.4 minutes). Although drug users and non-users reported similar sleep duration (about 7.4 hours), drug users showed poorer subjective sleep quality and habitual sleep efficiency. They reported more sleep disturbance and need for sleep medications, more daytime dysfunction and poorer subjective sleep quality compared with nondrug users. The total PSQI score positively correlated with the duration of drug use (rp = 0.164, p < 0.001). We also found a link between sleep problems and cigarette smoking, alcohol drinking, and duration of drug use.

**Conclusions:**

Poor sleep quality is common among illicit drug dependent subjects. Long-term substance users had more sleep problems. Future research aiming at quantifying the benefits of treatment interventions should not neglect the influence of sleep problems. Gaining more insight into the impact of sleep quality on the addiction treatment could also help to target future intervention measures more effectively.

## Background

Illicit drug abuse and drug addiction have been recognized as major problems such as the increased risk of spreading HIV and other blood-borne viruses, drug-related criminal activities, drug-related financial problems, family issues, and public healthcare expenditures in our society. Drug abuse has been recognized as a major problem in China since the 1980s and has quickly and dramatically increased during the past decade [1]. Although the actual number of drug addicts in China is not known, it has the world’s largest population of illicit drug users [1,2].

Sleeping is one of essential maintenance activities in every individual’s life. Sleep problems may lead to physical and mental disorders, and even social disturbances. For example, poor sleep quality has been associated with treatment-resistant hypertension [3] and suicidal ideation [4]. Sleep disturbances are common in the general population [5,6], and more pronounced in drug-using populations [7-9]. Preclinical studies suggest that chronic sleep deprivation had effects on drug-seeking and drug-taking behaviors. It also had an effect on the willingness to work for drugs, and greatly enhanced response for cocaine in rats [10]. Similarly, clinical studies suggest that poor sleep quality was associated with increased frequency of drug use and relapse [8]. Several studies have reported sleep problems associated with the use of alcohol [11-14]. There were also some studies of sleep problems in opioid use and methadone maintenance subjects. Assessing by the Pittsburgh Sleep Quality Index (PSQI), our preliminary study with 140 Chinese heroin-dependent patients found that the majority of them reported poor sleep quality (96.30% had PSQI ≥ 8) [7]. 96.6% of the drug users in a 30 subjects sample study reported a total PSQI score of 6 or higher, suggestive of poor quality of sleep [15]. 70.2% of patients had PSQI scores >5 in a 121 heroin addicts who were receiving MMT sample [16], compared with those methadone maintenance treatment (MMT) patients who had history of longer duration of opiate usage and a shorter period in MMT with no methadone take-home doses (THD) (80.6%, PSQI > 5). MMT patients who had two weeks achievement of THD showed lower rates of poor sleep (56%, PSQI > 5) [17].

According to 2013 National Drug Abuse Monitoring Annual Report, the abuse of traditional drugs (mainly heroin) has been decreased recently. But the abuse of synthetic drugs (mainly methamphetamine and ketamine) showed a rising trend (http://www.sda.gov.cn/WS01/CL1404/107039.html, Chinese). However, little work has been done on investigating sleep quality in stimulants (mostly methamphetamine, also called “ice”) and ketamine dependent subjects. The present study aimed to investigate the frequency of poor sleep quality among 2178 illicit drug dependent subjects and 2236 non drug users, and to assess the correlation between sleep quality as well as duration of drug use. Based on previous studies, we hypothesized that most illicit drug dependent subjects would have poorer sleep quality, and prevalence of poor sleep quality subjects will be much higher in illicit drug users when compared with non drug using controls. Duration of substance abuse may have impact on quality of sleep. Peles E found that subjects with a longer duration of opiate usage had poorer sleep [17]. Pasch KE Found a longitudinal bi-directional relationships between sleep quality and addictive substance use (such as cigarettes, alcohol or marijuana) [18]. Furthermore, our previous study showed that duration of ketamine use has been associated with alteration of brain structure [19]. Thus, we also hypothesized that self-reported poor sleep quality would be associated with the longer duration of substance use.

## Methods

### Design

A cross-sectional descriptive survey design was used to explore sleep quality in illicit drug (including marijuana) dependent subjects in two drug rehabilitation centres in Changsha, China.

### Subjects

Participants were recruited from November 2010 to April 2014 in Changsha city, Hunan province, China. We recruited 2315 illicit drug dependent subjects from two drug rehabilitation centres (1949 subjects came from the Kangda Voluntary Drug Rehabilitation Centre in Hunan Province and 366 subjects came from the Department of Addiction Medicine, Hunan Brain Hospital) and 2390 healthy nondrug users with negative illicit drug use test through a combination of targeted site sampling, advertisement and snowball sampling referrals. Among them, 2178 drug users (1824 subjects came from the Kangda Voluntary Drug Rehabilitation Centre in Hunan Province and 354 subjects came from the Department of Addiction Medicine, Hunan Brain Hospital) and 2236 nondrug users from seven communities in Hunan province completed all questions. Sampling of nondrug users was collected by visiting demographically matched households. The data of illicit drug dependent subjects was collected following completion of a 10-day detoxification-treatment. All these drug use subjects met the Diagnostic and Statistical Manual of Mental Disorders, fourth edition criteria for lifetime ketamine dependence determined from the Structured Clinical Interview. Control subjects were included only if they had never used any drugs. Control subjects were excluded if they reported major medical or psychiatric disorders.

### Assessment measures

In addition to some demographic information, self-report measure of Pittsburgh Sleep Quality Index (PSQI) [20] was used. Since its introduction in 1989, the PSQI had been widely used and well-validated measure of sleep quality in various cultures. It is a self-rating questionnaire used to assess subjective sleep quality during the previous month. It contains 19 self-rated questions, resulting in both a global score and 7 component sub-scores: subjective sleep quality, sleep latency, sleep duration, sleep efficiency, sleep disturbance, use of sleep medication and daytime dysfunction. Each component has been scored from 0 to 3, resulting in global PSQI score between 0 and 21, with higher scores indicating a lower quality of sleep. A PSQI global score greater than 5 indicates ‘poor sleep’ with a sensitivity of 89.6–98.7% and specificity of 84.4–86.5% [20,21]. Also, there has a suggestion of a cut-off of the global score at higher than 8, which means a severe sleep problem [22,23]. The questionnaire is easy to handle and can be completed within 5 min. The Chinese language version of PSQI is well validated, reliable and widely used in patients and general populations [24,25].

### Procedure

Before conducting the study, both the study and oral informed consent were approved from the Second Xiangya Hospital of Central South University Institutional Review Board (No.S117, 2009). Drug dependent participants have been invited to answer the short self-reported questionnaire on the first week of the administration. As part of the consent process, participants were provided with a detailed explanation of the objectives of the study and study expectations. Participants were advised of their ability to withdraw from the study at any point without penalty or adverse consequences. Issues of confidentiality and anonymity were discussed. Oral informed consent was obtained. Participants were encouraged to answer the questionnaire independently and as soon as possible.

### Statistical analysis

Statistical analysis was performed using the SPSS for Windows (Version 16, SPSS Inc., Chicago, IL, USA) software package. To address missing data, incomplete questionnaires were excluded. Descriptive statistics were used to examine demographic characteristics and pooled responses. Independent sample t-tests were conducted to determine group differences (drug use and nondrug use groups) in sleep problems. Correlation between PSQI total scores and duration of illicit drug use was explored using the Pearson correlation coefficients. Multiple linear regression model has been applied to evaluate the impact of age, gender, number of cigarettes smoked per day, drinking or not, duration of drug use on Sleep problems. An alpha level of .05 was set to determine statistical significance.

## Results

### Sample characteristics

2178 subjects in 2315 (94.08%) drug users and 2236 subjects in 2390 (93.56%) nondrug users agreed to participate in the study and completed all questions. The overall response rate was 93.82%. The overall sample group was characterized typically by middle class socioeconomic status. The two groups were age and gender-matched. Demographic characteristics of illicit drug users and control subjects are shown in Table 1.

**Table 1:** **Demographic, cigarette smoking and alcohol drinking characteristic of participants**

		2178 Drug users	2236 Nondrug users
Age, M ± SD		30.04 ± 7.134	30.58 ± 11.145
Educational levels, M ± SD*		10.63 ± 2.975	12.10 ± 3.225
Gender (n, %)	Male	1875, 86.1%	1910, 85.4%
	Female	303, 13.9%	326, 14.6%
Ethnicity (n, %)	Han Chinese	2149, 98.8%	2156, 96.5%
	Minorities	26, 1.2%	79, 3.5%
Employment status (n, %)	Employed	1214, 56.0%	1360,61.1%
	Unemployed	953, 44.0%	866, 38.9%
Marital status (n, %)	Married	942, 43.3%	1136, 51.5%
	Unmarried	1227, 56.6%	1071, 48.5%
Cigarette smoking (n, %)	Smokers	2104, 96.7%	895, 40.3%
	Nonsmokers	71, 3.3%	1328, 59.7%
Alcohol drinking (n, %)	Drinkers	758, 34.9%	686, 30.9%
	Nondrinkers	1413, 65.1%	1535, 69.1%

M: mean; SD: standard deviation; n: number; %: the percentage of subjects.

Cigarette smoker was defined as smoked more than 100 cigarettes in the life time.

Alcohol drinking was defined as drunk no less than 30 g alcohol (equal to 900 ml beer) per week.

^*^ Significantly different from control group, p < 0.01.

### Drug use characteristics

46.5% (1012) mainly heroin users, 44.2% (962) mainly methamphetamine users, 7.9% (172) mainly ketamine users, and 1.5% (32) other drug users (including 18 ‘Ma Gu’ (amphetamine and caffeine), 5 marijuana users, 3 cocaine users, 2 “HuYouyou” (Methaqualone and ephedrine), 2 tramadol hydrochloride, 1 dihydroetophine user and 1 “GHB” (Gamma hydroxybutyrate) user). Most of “club-drug” users were poly-substance users (used methamphetamine, ketamine, and ‘Ma Gu’). All of the drug users met criteria for at least one substance abuse or dependence according to the Diagnostic and Statistcal Manual for Mental Disorders, Fourth Edition (DSM-IV) criteria. The mean duration of drug use (months) was 59.5 ± 52.26 (heroin users: 82.1 ± 60.81, methamphetamine users: 36.2 ± 31.44, ketamine users: 56.4 ± 31.319, others drug users: 56.3 ± 38.05). The quantity and frequency of heroin, methamphetamine and ketamine use in the last three month before administration was shown as follow: heroin users used 1.72 ± 0.98 g heroin and 1.06 ± 0.34 times per day; methamphetamine users used 0.61 ± 0.91 g methamphetamine and 2.27 ± 1.13 times per day; ketamine users used 1.37 ± 1.38 g ketamine powder and 1.53 ± 0.94 times per day.

### Sleeping problems

Analysis of the PSQI revealed that the mean Sleep latency (defined as the amount of time it takes to fall asleep after the lights have been turned off) was 37.7 ± 38.624 minutes in drug users and 18.4 ± 23.578 minutes in nondrug users (p < 0.001); the mean total sleep time was 7.4 ± 2.140 hours in drug users and 7.3 ± 1.278 hours in nondrug users (p = 0.101), which is much the same as the widely accepted norm of 7–8 hours of sleep per night. Detailed information for score of the PSQI components and PSQI total score between drug users and nondrug users is shown in Table 2. The drug users had higher scores than nondrug users for seven components and total scores of PSQI. Daytime dysfunction, sleep latency and subjective sleep quality were the most severe sleep problems in drug users.

**Table 2:** **Score of the PSQI components and PSQI total score between drug users and nondrug users**

	Drug users	Non drug users	P
	(n = 2178)	(n = 2236)	
Subjective sleep quality, M ± SD	1.41 ± 0.897	0.75 ± 0.637	<0.001
Sleep latency, M ± SD	1.43 ± 1.128	0.73 ± 0.724	<0.001
≤15 min, n,%	568, 26.1%	914, 40.9%	-
16-30 min, n,%	660, 30.3%	1048, 46.9%	-
31-60 min, n,%	400, 18.4%	227, 10.2%	-
>60 min, n,%	550, 25.3%	47, 2.1%	-
Sleep duration, M ± SD	0.84 ± 0.999	0.74 ± 0.817	<0.001
>7 h, n,%	1095, 50.3%	1081, 48.3%	-
5–7 h, n,%	891, 40.9%	1118, 50%	-
<5 h, n,%	192, 8.8%	37, 1.7%	-
Habitual sleep efficiency*, M ± SD	0.67 ± 1.048	0.30 ± 0.689	<0.001
≥85%, n,%	1420, 65.2%	1779, 79.6%	-
75–84%, n,%	329, 15.1%	305, 13.6%	-
65–74%, n,%	167, 7.7%	81, 3.6%	-
<65%, n,%	262, 12.0%	71, 3.2%	-
Sleep disturbance, M ± SD	1.12 ± 0.735	0.70 ± 0.536	<0.001
Need for sleep medications, M ± SD	0.88 ± 1.241	0.06 ± 0.281	<0.001
Not during the past month, n,%	1359, 62.4%	2129, 95.2%	-
Less than once a week, n,%	173, 7.9%	88, 3.9%	-
Once or twice a week, n,%	185, 8.5%	15, 0.7%	-
≥3 times a week, n,%	461, 21.2%	4, 0.2%	-
Daytime dysfunction, M ± SD	1.63 ± 1.109	0.91 ± 0.848	<0.001
PSQI total score, M ± SD	7.97 ± 4.392	4.20 ± 2.468	<0.001
Good sleepers (PSQI ≤ 5), n,%	686, 31.5%	1645, 73.6%	-
Poor sleepers (PSQI > 5), n,%	1492, 68.5%	591, 26.4%	-

M: mean; SD: standard deviation; n: number; %: the percentage of subjects.

Mean differences were analyzed between drug users and non drug users by t-tests; Score ranges from 0 to 3, with higher scores indicating poorer functioning. Habitual sleep efficiency^*^ = total hours of sleep/(get-up time − bedtime) × 100%.

Subjects were divided into groups of good sleepers (PSQI ≤ 5) and poor sleepers (PSQI > 5). A majority of the drug abuse subjects (68.5%) and slightly more than one fourth (26.4%) nondrug users had a total PSQI score of 5 or higher, suggestive of mild sleeping problems; 43.9% (956) drug use subjects and 5.2% (117) nondrug users had PSQI >8, suggestive of poor quality of sleep. Sleeping quality for difference types of drug users is shown in Table 3.

**Table 3:** **Sleeping quality for difference types of drug users**

Drugs of users	n,%	PSQI total score, M ± SD	PSQI > 5	PSQI > 8
			n,%	n,%
heroin	1012, 46.5%	9.18 ± 4.047	812, 80.24%	559, 55.24%
“ice”	962, 44.2%	6.60 ± 4.476	521, 54.16%	314, 32.64%
ketamine	172, 7.9%	8.75 ± 3.762	141, 81.98%	79, 45.93%
others	32, 1.5%	7.03 ± 3.478	19, 59.36%	7, 21.88%

M: mean; SD: standard deviation; n: number; %: the percentage of subjects. “ice”: methamphetamine.

### Sleep problems and duration of drug use (months)

Pearson correlation analyze was conducted for the correlation of the total PSQI score and the duration of illicit drug use (months). The total PSQI score positively correlated with the duration of drug use (rp = 0.164, p < 0.001).

### Sleep problems among cigarette smokers and nonsmokers, alcohol drinkers and nondrinkers

*T* test has been applied for assessing the differences between cigarette smokers and nonsmokers, alcohol drinkers and nondrinkers among drug users and nondrug users. In the nondrug use group, both smokers and alcohol drinkers had relatively high PSQI total score. However, in the drug use group, both smokers and alcohol drinkers had relatively low PSQI total score (see Table 4).

**Table 4:** **The PSQI total score between smokers and nonsmokers, drinkers and nondrinkers among drug users and non users**

Variables			PSQI total score		
			n	M ± SD	p
Non users	Smoking	Yes	895	4.38 ± 2.583	0.004
		No	1328	4.07 ± 2.378	
	Alcohol drinking	Yes	686	4.57 ± 2.463	<0.001
		No	1535	4.03 ± 2.449	
Drug users	Smoking	Yes	2104	7.94 ± 4.427	0.01
		No	71	8.94 ± 3.112	
	Alcohol drinking	Yes	758	6.99 ± 5.027	<0.001
		No	1413	8.51 ± 3.916	

n: number; M: mean; SD: standard deviation.

### Variables (age, gender, number of cigarettes smoked per day, drinking, duration of drug use) predicting increased Sleep problems

Multiple linear regression models have been applied to evaluate the impact of age, gender, number of cigarettes smoked per day, drinking or not, duration of drug use on Sleep problems. It showed that age, gender, number of cigarettes smoked per day, drinking or not, duration of drug use were related to PSQI total score. In other words, increased age, female, cigarette smokers who smoked more cigarettes per day, alcohol drinkers, drug users with longer-term drug use had more sleep problems (see Table 5).

**Table 5:** **MLR model predicting increase in PSQI total score**

Variable	B	95% Confidence interval	p
Age	0.048	0.015 to 0.080	0.004
Gender	1.126	0.422 to 1.830	0.002
Cigarette/day	0.032	0.013 to 0.051	0.001
Drinking or not	0.736	0.264 to 1.209	0.002
Duration of drug use (M)	0.011	0.006 to 0.016	<0.001

Multiple linear regression model (MLR) model.

Y =4.555 + 0.048 age + 1.126 gender + 0.032 cigarette/day + 0.736 drinking + 0.011 duration of drug use (Months).

## Discussion

We found, as hypothesized, that the prevalence of sleep disturbance was much higher in drug users (68.5%, PSQI >5) than nondrug users (26.4%, PSQI >5), and that those drug users who experienced longer duration of drug use had poorer sleep quality. In addition, this study found that drug users (37.7 minutes) had approximate twice sleep latency than nondrug users (18.4 minutes). Although drug users and nondrug users reported similar sleep duration (about 7.4 hours), drug users showed poorer subjective sleep quality and habitual sleep efficiency, more sleep disturbance and need for sleep medications, more daytime dysfunction and poorer subjective sleep quality compared with nondrug users. Furthermore, we found that the total PSQI score positively correlated with the duration of drug use (rp = 0.164, p < 0.001); increased age, female, cigarette smokers who smoked more cigarettes per day, alcohol drinkers, drug users with longer-term drug use were exhibiting high PSIQ total score (more sleep problems).

### Sleep problems in illicit drug users

This study showed that a majority of the drug users (68.5%) had PSQI >5 (suggestive of mild sleeping problems), which was much higher compared with non drug users (26.4%); almost half of the drug users (43.9%) had PSQI >8 (suggestive of poor quality of sleep), and only 5.2% of nondrug users had PSQI >8. High prevalence of sleep disturbance in this study is consistent with previous studies. For example, Peles et al. found that 75.2% of the 101 methadone maintenance treatment patients reported prevalence of sleep disorders (PSQI >5) [26]; in a cohort of 225 methadone maintenance treatment patients. Stein et al. found 84% of them reported sleep disturbance (PSQI >5) [27]. Our previous pilot study found that 134 heroin dependent patients reported sleeping problems (PSQI >5) among135 subjects [7].

Among the seven components, daytime dysfunction, subjective sleep quality, and sleep latency were the most severe sleep problems in drug users. However, Drug users (0.84 ± 0.999) and nondrug users (0.74 ± 0.817) in this study reported less significant differences in sleep duration. The similarity of sleep duration between drug users and nondrug users contradicted with previous reports and indicates a need further studies. Sharkey et al. reported that average sleep times for the diary, morning questionnaire, and polysomnography in opioid dependence were 340, 323, and 332 min, respectively [28]. Chaputa et al. reported that, when participants were categorized as short- (⩽6 h), average- (7–8 h) or long- (>8 h) duration sleepers, short-duration sleepers consumed significantly more alcohol than the two other sleep-duration groups [29]. A study from Chakravorty et al. also showed a link between decreased sleep duration and heavy alcohol consumption [30]. Palmer et al. found a strong negative association between cigarette smoking and sleep duration on both men and women, and between alcohol consumption and sleep duration in men [31]. Smokers also showed shorter sleep period time [32].

Heroin users had longest mean of duration of drug use and showed the highest mean of PSQI total score, followed by ketamine users and users used other drugs. Compared with them, methamphetamine users used it for a relatively short period and showed relatively low total PSQI score. Furthermore, Pearson correlation analyze was also showed a positive correlation between total PSQI score and the duration of illicit drug use (months), which indicated that long-term substance users had more sleep problems. Linear regression analyze also showed that drug users with longer-term drug use is associated with more sleep problems. Lifetime drug use and drug use disorder may be a proxy for other variables like other chronic maladaptive behaviors, prior trauma, antisocial, borderline and schizotypal personality disorders [33].

### Impact of alcohol drinking and cigarette smoking on sleep

We found that cigarette smokers who smoked many cigarettes per day and alcohol drinkers had more sleep problems. Cigarette smoking and alcohol drinking are commonly comorbid with illicit drug use disorders and may aggravate sleep disturbance [34]. The association found between sleep problems and alcohol drinking and cigarette smoking in our study is consistent with prior literature. Disturbed sleep in alcohol drinkers was widely studied [35,36]. Alcohol dependent patients who were smokers showed greater difficulty falling asleep [34]. Sleep disturbance is also common among cigarette smokers [37,38] and predicts smoking cessation failure [39]. A survey with 484 individuals showed that compared with nonsmokers, cigarette smokers reported more problems going to sleep, problems staying asleep, and daytime sleepiness [40]. There is also an association between adolescent smoking and sleep disorders [38] Our study found an association between number of cigarettes smoked per day and sleep problems (smoked more cigarettes per day had more sleep problems). However, Riedel et al. found light smoking (<15 cigarettes per day), but not heavier smoking, was associated with self-reported sleep problems [41]. Further studies are needed to clarify the relationship between total number of cigarettes smoked per day and sleep quality.

### Sleep problems and clinical implication

The co-occurrence of sleep problems and drug abuse is clinically significant because previous studies proved that sleep problems among drug abuse patients have been associated with subsequent drug relapse [42,43]. Disturbed sleep is an important predictor of relapse. A line of this study showed that sleep problems among alcoholic patients have been significantly associated with subsequent relapse [11,44]. Self-reported sleep problems can provide clinicians with information to plan better treatment for alcohol abusers [13]. Despite the evidence that alcohol-related sleep disturbances are predictors of relapse, this area of illicit drug abuse research is still in its infancy. Since sleep disturbance may as a predictor of treatment outcome, investigating their role in drug abuse may be particularly advantageous in furthering our knowledge about predicting addiction treatment outcome. Prospective studies are needed in order to verify the correlation between sleep problems and relapse, such as whether sleep disturbances during illicit drug withdrawal predict treatment outcome. Furthermore, impaired sleep may also contribute to reduced cognitive function [45].

### Strengths and limitations of the study

The sample size is relatively large in this study, and the control group was age and gender matched to the group of drug users. However, the current study only focused on sleep status, other factors that can have impact on sleep quality have not been considered in the study. Other limitations including collection of data from two drug rehabilitation centers (it may not be representative of all illicit drug users) with brief self-administered questionnaires; lack of objective sleep metrics. All illicit drug categories have been addressed as one; duration of drug use (months) may be associated with a recall bias. Also, the controls were recruited from snowball sampling in seven communities, and the controls were not matched for factors as mental health, and prescribed drugs. Future work should use the PSQI in longitudinal studies with more clinical factors and objective sleep metrics, and special attempts should be made to recruit larger samples targeting female drug use subjects to further elucidate the findings of this study.

## Conclusion

In conclusion, we found that the prevalence of sleep disturbance was much higher in drug users than nondrug users, and that those drug users who had longer duration of drug use showed poorer sleep quality. In addition, this study found that drug users had approximately twice the sleep latency that nondrug users exhibited. Although drug users and non-users reported similar sleep duration, drug users showed poorer subjective sleep quality and habitual sleep efficiency. They also exhibited more sleep disturbance and need for sleep medications, more daytime dysfunction and poorer subjective sleep quality compared with nondrug users. We also found an association between sleep problems and cigarette smoking, alcohol drinking, and duration of drug use. This study exemplifies the high prevalence of poor sleep quality in illicit drug dependent subjects. Future researches aim at quantifying the benefits of treatment interventions should not neglect the influence of sleep problems. Gaining more insight into the impact of sleep quality on the addiction treatment could also help to target future intervention measures more effectively.
